# A review of advances in research on mechanisms inhibiting kidney stone formation from a network pharmacology perspective

**DOI:** 10.3389/fphar.2026.1872382

**Published:** 2026-09-29

**Authors:** Xuming Zhang, Chaoyue Ji, Weiguo Hu

**Affiliations:** Department of Urology, Beijing Tsinghua Changgung Hospital, School of Clinical Medicine, Tsinghua Medicine, Tsinghua University, Beijing, China

**Keywords:** clinical translation, critical appraisal, global ethnomedicine, kidney stone disease, network pharmacology, pathophysiology, pharmacokinetics, phytochemical diversity

## Abstract

Nephrolithiasis is a prevalent, highly recurrent urological disorder affecting 1%–15% of the global population, with 5-year recurrence rates reaching up to 50%. While surgical interventions achieve acute stone clearance, they do not resolve underlying metabolic derangements, crystal-induced tubular injury, or chronic inflammatory cascades. Natural products from global ethnomedicinal systems (including Traditional Chinese Medicine, Indian Ayurveda, Persian Unani medicine, European phytotherapy, and African ethnopharmacology) demonstrate potent multi-target anti-urolithic properties. However, decoding their intricate polypharmacology remains a major challenge. Network pharmacology, combining multi-omics profiling, computational target prediction, and network topology, has emerged as a promising paradigm to decipher these multi-component mechanisms. Unlike previous descriptive reviews that largely focused on narrow botanical catalogs or local traditions, this review establishes a critical, globally inclusive, and methodologically rigorous synthesis of network pharmacology-driven nephrolithiasis research from 2022 to 2026. We detail the fundamental pathophysiology across major stone types (calcium oxalate, calcium phosphate, uric acid, struvite, cystine) and the comprehensive cellular cascade encompassing crystal nucleation, Randall’s plaque/plug formation, CD44-mediated adhesion, mitochondrial reactive oxygen species burst, endoplasmic reticulum stress, NLRP3 inflammasome activation, macrophage M1/M2 polarization, immunometabolism, extracellular vesicle communication, and programmed cell death (apoptosis, necroptosis, pyroptosis, ferroptosis, and PANoptosis). We critically synthesize evidence across diverse chemical classes (flavonoids, polyphenols, pentacyclic triterpenes, alkaloids, anthraquinones, tannins, polysaccharides, and marine bioactive metabolites), bridging computational predictions with established mechanistic milestones. Furthermore, we provide a deep critical appraisal of core methodological bottlenecks—target prediction bias, publication bias of over-studied hub genes, arbitrary pharmacokinetic cutoffs, circular virtual docking, and lack of renal bioavailability validation. Finally, we formulate a forward-looking translational roadmap integrating single-cell transcriptomics, humanized 3D organoid co-cultures, spatial multi-omics, reverse validation in longitudinal clinical registries, and machine learning feature selection, providing rigorous guidance for precision anti-urolithic drug discovery.

## 1 Introduction

Kidney stone disease (nephrolithiasis or urolithiasis) represents an escalating global health burden affecting approximately 1%–15% of the worldwide population, with incidence and prevalence rates rising steadily over recent decades across diverse geographical regions (Wang Z. et al., 2021; Jong et al., 2025). Beyond causing excruciating acute renal colic and urinary obstruction, recurrent nephrolithiasis is intrinsically linked to progressive renal parenchymal injury, chronic kidney disease (CKD), end-stage renal disease (ESRD), and elevated cardiovascular morbidity (Jong et al., 2025; Antonelli et al., 2014). Contemporary clinical management predominantly relies on surgical interventions—including percutaneous nephrolithotomy (PCNL), flexible ureteroscopic lithotripsy (URS/RIRS), and extracorporeal shock wave lithotripsy (ESWL) Srivastava et al., 2025. While these techniques achieve high initial stone-free rates, they remain inherently symptomatic interventions that carry perioperative risks (e.g., hemorrhage, sepsis, ureteral strictures) and fail to correct the underlying metabolic disturbances or molecular drivers of crystallization (Srivastava et al., 2025; Lai et al., 2022). Consequently, 5-year recurrence rates remain as high as 30%–50%, underscoring an urgent clinical need for efficacious, safe pharmacological agents for long-term stone prevention and recurrence control (Lai et al., 2022; Khan et al., 2016; Ahmed et al., 2018; Lobine et al., 2020)

A major obstacle in anti-urolithic drug development is the multifaceted etiology and intricate pathophysiology governing biomineralization. Mineralogically, kidney stones are classified into five primary categories (Khan et al., 2016; Sorokin et al., 2017): (1) Calcium oxalate (CaOx) stones (∼70%–80%), primarily as calcium oxalate monohydrate (COM, whewellite) and calcium oxalate dihydrate (COD, weddellite); (2) Calcium phosphate (CaP) stones (∼10%–15%), including carbonate apatite and brushite, frequently associated with distal renal tubular acidosis, primary hyperparathyroidism, or alkaline urine (pH > 6.5); (3) Uric acid (UA) stones (∼8%–10%), driven by hyperuricosuria, metabolic syndrome, and overly acidic urine (pH < 5.5); (4) Infection-induced or struvite stones (∼1%–5%), composed of magnesium ammonium phosphate precipitated by urease-producing bacteria (e.g., *Proteus mirabilis*); and (5) Cystine stones (∼1%), an autosomal recessive defect in renal dibasic amino acid reabsorption (SLC3A1/SLC7A9 mutations) (Khan et al., 2016; Sorokin et al., 2017).

The pathophysiological cascade involves a sequential biomineralization and cell-injury process (Khan et al., 2016; Evan et al., 2003; Xu et al., 2023). It initiates when urinary supersaturation (SS > 1) exceeds metastable limits, driving crystal nucleation, growth, and aggregation. In idiopathic CaOx formers, subepithelial calcification originates at the thin loops of Henle basement membranes, forming Randall’s plaques, whereas in brushite or acidosis formers, crystals plug the terminal collecting ducts (Randall’s plugs) (Evan et al., 2003; Khan et al., 2021). Crystal contact with tubular epithelial cells engages apical receptors (CD44, hyaluronan, annexin A2, osteopontin), triggering endocytosis and membrane destabilization. Intracellular crystal internalization provokes mitochondrial membrane potential collapse, massive reactive oxygen species (ROS) bursts, and endoplasmic reticulum (ER) stress (Khan et al., 2016; Xu et al., 2025a; Chaiyarit and Thongboonkerd, 2020). This stress milieu triggers assembly of the NLRP3 inflammasome, activating caspase-1 and releasing IL-1β and IL-18. Damaged tubular cells secrete chemokines (MCP-1/CCL2) that recruit monocytes and polarize intrarenal macrophages toward a pro-inflammatory M1 phenotype while suppressing reparative M2 polarization. Furthermore, crystal-induced stress activates multiple regulated cell death pathways—including apoptosis, necroptosis, pyroptosis, ferroptosis (driven by lipid peroxidation and GPX4 exhaustion), and PANoptosis—while extracellular vesicles (EVs) propagate pro-inflammatory signals to neighboring parenchyma, culminating in tubular denudation and progressive tubulointerstitial fibrosis (Khan et al., 2016; Xu et al., 2025a; Xu et al., 2024a).

To counter this complexity, natural bioactive products from global ethnomedicinal systems—including Traditional Chinese Medicine (TCM), Indian Ayurveda, Persian and Middle Eastern Unani phytomedicine, European phytotherapy, and African ethnopharmacology—offer potent multi-target anti-urolithic activities (Liang et al., 2024; Yao et al., 2024). However, deciphering their polypharmacology has historically been constrained by chemical heterogeneity. In recent years, network pharmacology—grounded in systems biology, multi-omics, and network topology—has emerged as a primary computational tool to decode these mechanisms (Hou et al., 2025; Li et al., 2026). Distinct from previously published reviews that have either descriptively cataloged specific regional herbs or focused narrowly on static computational outputs without critical methodological appraisal, this review establishes a comprehensive, globally inclusive, and critically analytical synthesis of network pharmacology-driven nephrolithiasis research from 2022 to 2026. Specifically, this review addresses critical literature gaps by: (1) Expanding the chemical and ethnomedicinal coverage beyond TCM to encompass Indian Ayurvedic, Persian, Middle Eastern, European, and African phytomedicines, as well as emerging marine-derived bioactive metabolites; (2) Bridging *in silico* predictions with established mechanistic milestones (such as polyphenol and pentacyclic triterpene pharmacology reviewed by Ahmed et al., 2018 and Lobine et al., 2020); (3) Providing an uncompromising critical appraisal of prevailing network pharmacology limitations—including target prediction bias, publication bias of over-studied hub genes, arbitrary pharmacokinetic cutoffs, circular virtual docking, and lack of renal bioavailability validation; (4) Establishing a structured evidence-grading framework distinguishing *in silico*, *in vitro*, *in vivo*, and clinical validation tiers; and (5) Formulating an actionable translational roadmap integrating single-cell transcriptomics, humanized 3D organoid co-cultures, spatial multi-omics, reverse validation in longitudinal clinical registries, and machine learning feature selection.

## 2 Application of network pharmacology in kidney stone research

### 2.1 PRISMA-informed literature search strategy and selection criteria

To ensure a transparent, reproducible, and rigorous synthesis, a structured literature retrieval adhering to PRISMA principles was executed across major international biomedical and chemical repositories, including PubMed/MEDLINE, Web of Science Core Collection, Embase, Cochrane Library, and China National Knowledge Infrastructure (CNKI), supplemented by specialized natural product databases (TCMSP, SymMap, ETCM, HERB, IMPPAT). The search strategy combined Medical Subject Headings (MeSH) and free-text keywords using Boolean logic.

The search timeframe focused on peer-reviewed articles published between January 2022 and early 2026, while foundational mechanistic reviews were retained for biological contextualization. Eligibility criteria were strictly applied: (1) Inclusion criteria: original research articles and systematic reviews applying network pharmacology to urolithiasis; studies evaluating defined natural monomers, purified botanical/marine fractions, or standardized polyherbal formulations; studies providing detailed target identification, pathway enrichment, and empirical experimental validation. (2) Exclusion criteria: duplicate publications and preprints lacking peer review; purely descriptive computational papers without biological plausibility; conference abstracts and non-peer-reviewed commentaries.

### 2.2 Methodological workflow and timeline evolution of network pharmacology (2015–2026)

Network pharmacology shifts drug discovery from the reductionist “one drug–one target” paradigm toward holistic “drug-target-pathway-disease” network mapping (Hou et al., 2025; Li et al., 2026; Maciejewska-Turska et al., 2025; Noor et al., 2022). The standard operational pipeline encompasses five sequential phases (Figure 1): (1) Bioactive constituent harvesting and pharmacokinetic filtering (OB ≥ 30%, DL ≥ 0.18, SwissADME); (2) Compound-target prediction (SwissTargetPrediction, SuperPred, PharmMapper); (3) Disease gene identification (GeneCards, OMIM, DisGeNET, GEO); (4) Intersection network construction and PPI topological analysis (STRING, Cytoscape); and (5) Functional enrichment (GO/KEGG) coupled with computational docking and experimental validation. Commonly utilized databases across these phases are compiled in Table 1.

**FIGURE 1:**
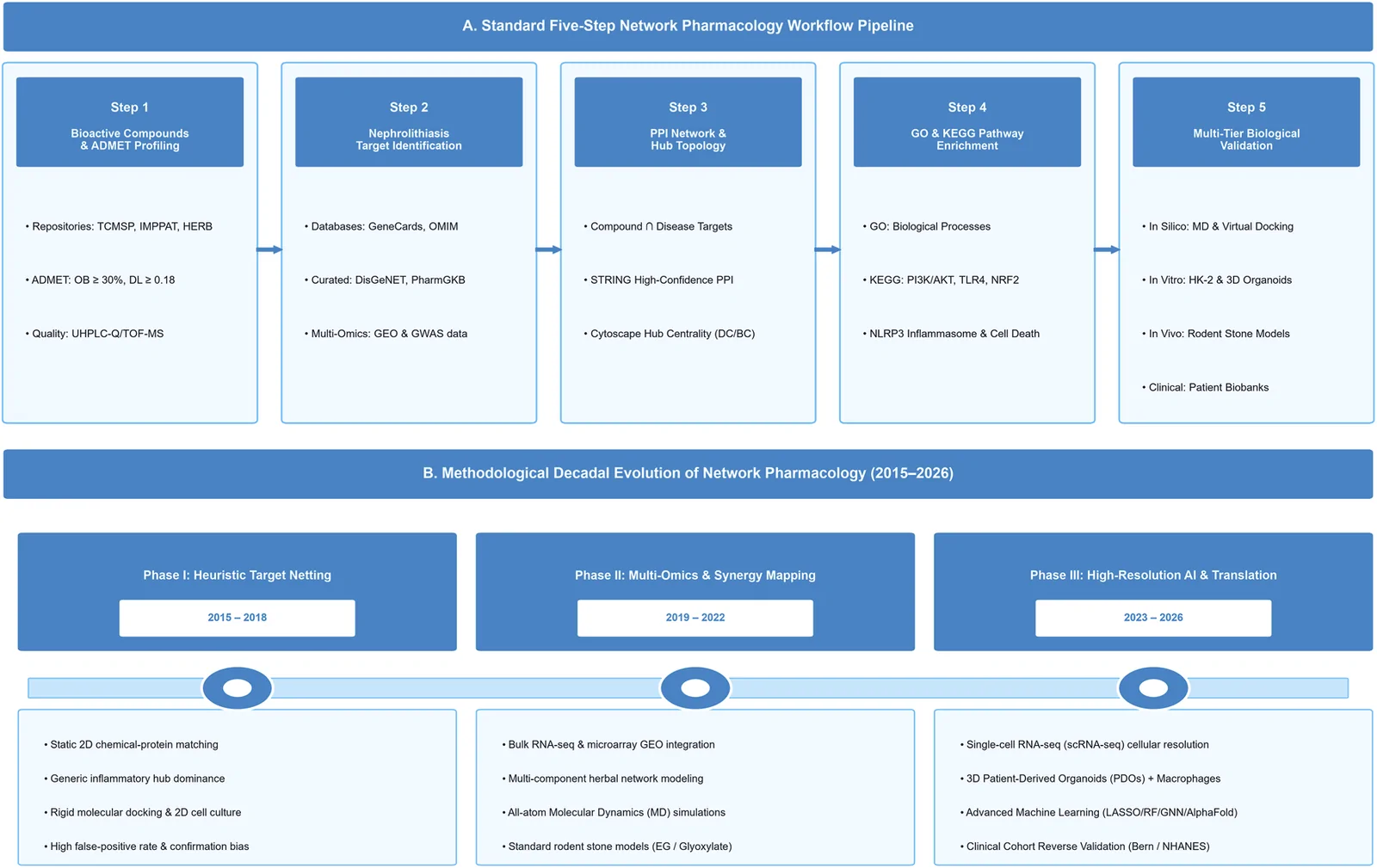
Standard operational workflow and decadal evolution (2015–2026) of network pharmacology in kidney stone disease research. **(A)** The standard pipeline integrates bioactive component harvesting, ADMET pharmacokinetic screening, disease target harvesting, PPI network construction, GO/KEGG functional enrichment, virtual docking, and multi-level experimental validation. **(B)** The timeline illustrates the methodological evolution from heuristic target netting (Phase I, 2015–2018) to multi-omics integration (Phase II, 2019–2022) and modern AI/machine learning-driven precision pharmacology (Phase III, 2023–2026).

**TABLE 1:** Commonly used databases and bioinformatics resources across analytical steps in nephrolithiasis network pharmacology research.

Analytical steps	Databases or websites	URL/description
Screen chemical composition and ADMET	HERB	http://herb.ac.cn
Screen chemical composition and ADMET	TCMSP	https://www.tcmsp-e.com/tcmsp.php
Screen chemical composition and ADMET	TCMIP	http://www.tcmip.cn/TCMIP/index.php
Screen chemical composition and ADMET	IMPPAT (Indian Medicinal Plants)	https://cb.imsc.res.in/imppat/
Screen chemical composition and ADMET	SwissADME	https://www.swissadme.ch
Screen chemical composition and ADMET	CTD	https://ctdbase.org
Screen chemical composition and ADMET	pkCSM	https://biosig.lab.uq.edu.au/pkcsm
Screen chemical composition and ADMET	Molsoft prediction tool	https://molsoft.com/mprop
Obtain chemical structures	HERB	http://herb.ac.cn
Obtain chemical structures	PubChem	https://pubchem.ncbi.nlm.nih.gov
Target prediction and identification	SwissTargetPrediction	https://www.swisstargetprediction.ch
Target prediction and identification	SuperPred	https://prediction.charite.de
Target prediction and identification	PharmMapper	http://lilab.ecust.edu.cn/pharmmapper
Target prediction and identification	UniProt	https://www.uniprot.org
Target prediction and identification	SEA Search Server	https://sea.bkslab.org/
Target prediction and identification	BATMAN-TCM	http://bionet.ncpsb.org/batman-tcm
Target prediction and identification	TargetNet	http://targetnet.scbdd.com
Target prediction and identification	DIGEP-Pred	http://www.way2drug.com/ge
Nephrolithiasis disease target harvesting	GeneCards	https://www.genecards.org
Nephrolithiasis disease target harvesting	PharmGKB	https://www.pharmgkb.org
Nephrolithiasis disease target harvesting	OMIM	https://www.omim.org
Nephrolithiasis disease target harvesting	DrugBank	https://go.drugbank.com
Nephrolithiasis disease target harvesting	Therapeutic Targets Database (TTD)	http://bidd.nus.edu.sg/group/ttd/ttd.asp
Nephrolithiasis disease target harvesting	DisGeNET	http://www.disgenet.org
Nephrolithiasis disease target harvesting	GEO (Gene Expression Omnibus)	http://www.ncbi.nlm.nih.gov/geo
Nephrolithiasis disease target harvesting	Phenolyzer	https://phenolyzer.wglab.org
PPI network construction and analysis	STRING	https://cn.string-db.org
PPI network construction and analysis	Cytoscape	https://cytoscape.org
PPI network construction and analysis	IID	https://iid.ophid.utoronto.ca
PPI network construction and analysis	GeneMANIA	https://genemania.org
PPI network construction and analysis	STITCH	http://stitch-db.org
Functional enrichment analysis	Metascape	https://metascape.org
Functional enrichment analysis	DAVID Bioinformatics Resources	http://david.ncifcrf.gov
Functional enrichment analysis	SangerBox	http://sangerbox.com

Over the past decade (2015–2026), network pharmacology in nephrolithiasis has undergone a pronounced methodological evolution across three distinct epochs: (1) Phase I (2015–2018, Heuristic Target Netting): Early studies focused on crude database intersections and static topological degree rankings, frequently producing uncurated target lists dominated by generic inflammatory hubs; (2) Phase II (2019–2022, Multi-Omics and Multi-Component Integration): The field transitioned toward combining bulk transcriptomics (GEO/RNA-seq) with molecular docking and dynamic simulations, validating multi-component herbal synergies in standard cellular and rodent models; (3) Phase III (2023–2026, High-Resolution, Humanized, and Data-Driven AI Pharmacology): Modern investigations increasingly leverage single-cell transcriptomics, spatial omics, humanized 3D patient-derived organoid (PDO) co-cultures, clinical biobank reverse validation, and machine learning feature selection (LASSO, Random Forest, GNNs), transforming predictive network models into rigorous translational engines.

### 2.3 Global ethnomedicine and phytochemical diversity in nephrolithiasis

Natural products against urolithiasis originate from diverse ethnomedicinal traditions across the globe. Below, we systematically synthesize their active constituents, molecular mechanisms, and evidence levels across major chemical families.

#### 2.3.1 Plant-derived bioactive monomers across diverse chemical classes

Network pharmacology coupled with molecular docking has accelerated the identification of botanical monomers exerting potent protective effects against calcium oxalate crystal-induced renal injury across multiple distinct chemical families (Table 2):

1. Polyphenols, Flavonoids, Coumarins, and Phenolic Glycosides: As comprehensively reviewed in landmark mechanistic literature by Ahmed et al. (2018), dietary polyphenols and flavonoids attenuate calcium oxalate urolithiasis through multi-target antioxidant, anti-inflammatory, and crystal-modulating mechanisms. Within network pharmacology pipelines, umbelliferone (coumarin) activates PI3K/AKT via PIK3CA, blunting excessive autophagy, ROS generation, and inflammatory cytokine surge (Xu et al., 2025a). Puerarin protects HK-2 cells against crystal-induced apoptosis and inflammation via PI3K/AKT signaling (Xu et al., 2024a). Pectolinarigenin directly binds HIF-1α, suppressing hypoxia-inducible inflammatory and oxidative cascades (Yao et al., 2024). Beyond classical pathways, Li et al. (2026) revealed that salidroside (phenolic glycoside from *Rhodiola rosea*) targets EGFR, NFE2L2, and MTOR to inhibit crystal-induced PANoptosis—a newly characterized inflammatory programmed cell death pathway orchestrating cross-talk among apoptosis, pyroptosis, and necroptosis. In HK-2 cells and glyoxylate-induced mice, salidroside dose-dependently downregulated cleaved-caspase-3/8, phosphorylated RIPK3, and GSDMD-N, preventing PANoptosome assembly. Silibinin (flavonolignan) was validated by Bhura and Gupta (2025) to restore tubular viability via EGFR and BCL2 modulation.
2. Pentacyclic Triterpenes, Phytosterols, and Steroidal Saponins: As critically synthesized by Lobine et al. (2020), pentacyclic triterpenes (e.g., lupeol, betulin, oleanolic acid, ursolic acid, celastrol) possess broad-spectrum anti-urolithiatic properties, suppressing crystal nucleation, upregulating renal antioxidant defenses, and preserving tubular architecture. In recent network pharmacology studies, Mohan et al. (2026) demonstrated that the natural phytosterol/triterpenoid stigmasterol mitigates COM crystal nucleation and modulates core inflammatory targets including CASP3, MAPK1, and calcium-sensing pathways. In parallel, Mohan et al. (2025) demonstrated that active phytocompounds from Pleurolobus gangeticus (*Desmodium gangeticum*) exhibit high binding affinity against the calcium-sensing receptor (CaSR) and oxalate oxidase (OxO), attenuating renal tubular calcification cascades. Among steroidal saponins, Liang et al. (2024) showed that dioscin specifically targets TLR4, inhibiting downstream TLR4/NF-κB signaling and blunting crystal-induced inflammatory injury.
3. Phenolic Acids, Esters, and Organic Acids: Li M. T. et al. (2022) identified ellagic acid as the principal active constituent of *Phyllanthus niruri* L., which binds directly to SQLE, SCD, and HMGCS1 to attenuate lipid accumulation and suppress “lipid nephrotoxicity” in tubular epithelial cells. Hou et al. (2025) integrated Mendelian randomization with network pharmacology, establishing that coffee-derived chlorogenic acid confers causal protection against urolithiasis by preventing SET complex dissociation, downregulating ANP32A and APEX1, and blunting TLR4/NF-κB-dependent NLRP3 inflammasome activation. Zhou et al. (2023) found that the microbial metabolite butyrate specifically downregulates CYP2C9 expression, markedly diminishing intracellular ROS overproduction and crystal adhesion.
4. Alkaloids, Anthraquinones, Tannins, Polysaccharides, and Carotenoids: Among alkaloids, berberine activates AMPK while suppressing NF-κB and TGF-β1, alleviating hyperoxaluria-induced tubular damage and interstitial crystal deposition. Natural anthraquinones (emodin, rhein) modulate the TGF-β1/Smad signaling axis, suppressing crystal-induced epithelial-mesenchymal transition (EMT) and tubulointerstitial fibrosis. Hydrolyzable tannins (punicalagin, corilagin) act as powerful scavengers of mitochondrial free radicals and directly inhibit heterogeneous COM crystal nucleation. Plant-derived polysaccharides (Plantago asiatica and Astragalus polysaccharides) coat crystal surfaces and tubular membranes, exerting negative electrostatic repulsion that impedes crystal aggregation and apical adhesion while upregulating NRF2/HO-1 antioxidant defenses. Furthermore, carotenoids (astaxanthin, lycopene) prevent mitochondrial permeability transition pore (mPTP) opening and inhibit NLRP3 inflammasome activation.

**TABLE 2:** Comprehensive comparative summary of natural bioactive compounds from global ethnomedicines investigated for kidney stone prevention and management.

Bioactive compound and biological source	Chemical class	Ethnomedicinal source	Kidney stone model	Principal targets and pathways	Proposed anti-urolithiatic mechanisms	Experimental validation and docking score	Evidence level and translational status
Salidroside (*Rhodiola rosea* L.)	Phenolic glycoside	TCM/Tibetan ethnomedicine	COM-induced HK-2 cells; glyoxylate mice	EGFR, NFE2L2, MTOR; caspase-3/8, RIPK3, GSDMD	Inhibits PANoptosis, prevents PANoptosome assembly, reduces tubular cell loss	Western blot, qPCR, histology; docking: 7.8 kcal/mol	Level II (*in vivo* and *in vitro*); preclinical lead requiring PK evaluation
Ellagic acid (*Phyllanthus niruri* L.)	Polyphenolic organic acid	Ayurvedic/global ethnopharmacology	COM-induced HK-2 cells; ethylene glycol rats	SQLE, SCD, HMGCS1, TP53; lipid metabolism	Attenuates lipid nephrotoxicity, suppresses intracellular lipid accumulation	Oil Red O, western blot; docking: 8.5 kcal/mol	Level II (*in vivo* and *in vitro*); widely used dietary supplement; RCTs ongoing
Stigmasterol (phytosterol/plant extracts)	Phytosterol/triterpenoid	Ayurveda/Global phytotherapy	COM crystal nucleation assay; *in vitro* renal models	CASP3, MAPK1, CaSR, PTGS2; calcium-sensing and MAPK	Inhibits crystal nucleation, modulates renal calcium-sensing and apoptotic signaling	Turbidity assays, crystal growth kinetics; docking: 8.9 kcal/mol	Level III (*in vitro* and *in silico*); high safety profile; prospective clinical trials warranted
Pleurolobus gangeticus active fraction	Isoflavonoid/pterocarpan fraction	Indian ayurvedic medicine (shalaparni)	*In silico* renal receptor models; COM crystallization	CaSR, oxalate oxidase (OxO); renal calcium homeostatic axis	Modulates calcium-sensing receptor, degrades oxalate, suppresses calcification	*In vitro* crystal dissolution assays; docking: 8.1 kcal/mol	Level III (*in vitro* and *in silico*); bioactive monomer isolation in progress
Umbelliferone (umbelliferae plants)	Coumarin/phenylpropanoid	Persian/Traditional medicine	COM-induced HK-2 cells; CaOx rat model	PIK3CA, AKT1, EGFR; PI3K/AKT signaling	Inhibits excessive autophagy, restores cell viability, suppresses ROS generation	Autophagic flux, ROS assays, TUNEL; docking: 7.2 kcal/mol	Level II (*in vivo* and *in vitro*); oral formulation optimization needed
Dioscin (*Dioscorea villosa* L.)	Steroidal saponin	TCM/North American phytotherapy	COM-treated HK-2 cells; hyperoxaluric mice	TLR4, MYD88, NF-κB p65; TLR4/NF-κB axis	Suppresses TLR4-driven inflammatory cascades and pro-inflammatory cytokine surge	TLR4 reporter assays, ELISA, western blot; docking: 8.6 kcal/mol	Level II (*in vivo* and *in vitro*); dosing window optimization required
Pectolinarigenin (Cirsium japonicum)	Flavonoid glycoside	East asian traditional medicine	COM-induced HK-2 cells; EG-induced mice	HIF-1α, VEGFA, SOD2; HIF-1α/ROS pathway	Directly binds HIF-1α, blunting hypoxia-inducible inflammatory and oxidative stress	Co-IP, western blot, crystal adhesion; docking: 8.2 kcal/mol	Level II (*in vivo* and *in vitro*); requires renal pharmacokinetic profiling
Berberine (*Coptis chinensis* Franch.)	Isoquinoline alkaloid	TCM/ayurvedic medicine	Oxalate-injured NRK-52E cells; glyoxylate rats	AMPK, NFKB1, TGFB1; AMPK/mTOR and TGF-β1/Smad	Activates AMPK, inhibits tubular EMT and crystal-interstitial deposition	Western blot, masson staining, urinary oxalate; docking: 8.4 kcal/mol	Level II (*in vivo* and *in vitro*); approved clinical agent; nano-delivery to boost renal PK
Thymoquinone (*Nigella sativa* L.)	Monoterpene quinone	Persian and middle Eastern medicine (unani)	Ethylene glycol hyperoxaluric rats; HK-2 cells	NFE2L2, SOD, GPX, NF-κB, iNOS; NRF2/ARE axis	Scavenges mitochondrial ROS, inhibits lipid peroxidation, reduces stone deposition	Histology, MDA/SOD assays, western blot; docking: 7.1 kcal/mol	Level II (*in vivo* and *in vitro*); extensive dietary use; pilot human trials reported
Lupeol (Crataeva nurvala Buch.-Ham.)	Pentacyclic triterpene	Ayurvedic medicine (varuna)	Glycolate-induced hyperoxaluric rats; *in vitro* COM	CASP3, BAX, SOD1, OPN; apoptotic and antioxidant axis	Normalizes urinary oxalate, preserves membrane integrity, blunts crystal deposition	Histopathology, urinary enzymes, crystal count; docking: 8.7 kcal/mol	Level II (*in vivo* and *in vitro*); strong candidate for standardized herbal formulation
Emodin (*Rheum palmatum* L.)	Anthraquinone	TCM/European herbal medicine	COM-induced HK-2 cells; Hyperoxaluric rats	TGFB1, SMAD2/3, FN1, COL1A1; fibrosis signaling	Inhibits tubular EMT, attenuates interstitial extracellular matrix accumulation	qPCR for α-SMA, sirius Red staining; docking: 7.9 kcal/mol	Level II (*in vivo* and *in vitro*); non-laxative derivatives required for long-term use
Punicalagin (*Punica granatum* L.)	Ellagitannin	Middle Eastern/mediterranean medicine	COM crystallization in synthetic urine; HK-2 cells	SOD1, CAT, NFE2L2, CASP3; NRF2 antioxidant axis	Scavenges mitochondrial ROS, inhibits crystal surface growth and aggregation	Dynamic light scattering, ROS flow cytometry; docking: 9.1 kcal/mol	Level III (*in vitro* and *in silico*); high antioxidant potency; gut metabolism profiling needed
Plantago asiatica polysaccharides	Plant polysaccharide	TCM/Asian traditional medicine	*In vitro* COM crystallization; HK-2 cell adhesion	CD44, OPN, HYAL1; cell-crystal adhesion receptors	Coats crystal facets, creates negative electrostatic barrier, prevents cell attachment	Flow cytometry for adhesion, zeta potential; docking: N/A (polymer)	Level III (*in vitro* and *in silico*); classical botanical anti-stone remedy; requires fractionation
Astaxanthin (*Haematococcus pluvialis*)	Marine/Algal Carotenoid	Marine biotechnology	COM-challenged tubular cells; EG-treated rats	SOD2, GPX4, NLRP3, IL1B; NLRP3 inflammasome axis	Inhibits mPTP opening, blunts lipid peroxidation, suppresses pyroptosis	JC-1 mitochondrial potential, western blot; docking: 8.3 kcal/mol	Level II (*in vivo* and in vitro); approved commercial nutraceutical; strong translational promise
Fucoidan (Brown marine algae)	Marine sulfated polysaccharide	Marine ethnomedicine	COM crystal-adhesion assay; Hyperoxaluric rats	CD44, TLR4, annexin A2; apical adhesion complex	Competitively binds CD44, blocks crystal endocytosis and membrane injury	Confocal microscopy, urinary calcium clearance; docking: 7.6 kcal/mol	Level II (*in vivo* and i*n vitro*); high safety profile; molecular weight standardization underway
Marine collagen peptides (fish skin)	Bioactive marine peptide	Marine biotechnology	*In vitro* metastable CaOx supersaturated solution	Ca2+ chelation sites, free radical scavenging hubs	Chelates free calcium ions, retards crystal nucleation, protects tubular matrix	Conductivity crystallization kinetics, MTT assay; docking: 7.4 kcal/mol	Level III (*in vitro* and *in silico*); Food-grade bio-peptide; suitable for dietary stone prevention

#### 2.3.2 Animal- and marine-derived bioactive metabolites in nephrolithiasis

Emerging evidence indicates that natural products from marine and animal origins possess distinct structural and biochemical advantages in kidney stone prevention. Marine sulfated polysaccharides, notably fucoidan extracted from brown seaweeds, exhibit potent anti-urolithic efficacy: owing to their dense sulfate ester groups, fucoidans competitively bind to the CD44 crystal-adhesion receptor and mask crystal growth sites, blunting CaOx attachment to renal tubular apical membranes. Bioactive marine collagen peptides derived from fish hydrolysates possess pronounced calcium-chelating and antioxidant capacities, effectively retarding homogeneous crystal nucleation in supersaturated urine. Sea cucumber glycosaminoglycans (GAGs) serve as exogenous analogs of human urinary macromolecular inhibitors (such as chondroitin sulfate and heparan sulfate), repairing damaged urothelial glycosaminoglycan protective layers. Additionally, marine omega-3 polyunsaturated fatty acids (EPA and DHA) modulate the intrarenal arachidonic acid cascade, blunting prostaglandin E2 (PGE2)-mediated hypercalciuria, while amino-acid derivatives like taurine protect against membrane lipid peroxidation and mitochondrial swelling. Integrating marine-derived bioactive metabolites into network pharmacology expands the chemical diversity of therapeutic candidates beyond traditional phytomedicines.

#### 2.3.3 Global phytomedicine traditions: ayurveda, Persian, European, and African ethnomedicine

Beyond Traditional Chinese Medicine, global ethnomedicinal systems provide extensive pharmacological troves for urolithiasis: (1) Indian Ayurvedic Medicine: Ayurvedic formulations such as Cystone and classical botanical drugs—including *Tribulus terrestris* (Gokshura), *Boerhavia diffusa* (Punarnava), and *Crataeva nurvala* (Varuna)—contain rich fractions of lupeol, quercetin, and saponins that enhance urinary volume, restore calcium/oxalate homeostasis, and downregulate intrarenal CASP3 and NF-κB cascades; (2) Persian and Middle Eastern Unani Medicine: Phytomedicines such as *Nigella sativa* (Black cumin), rich in thymoquinone, and *Alhagi maurorum* exert profound nephroprotective actions by scavenging mitochondrial superoxide, inhibiting renal iNOS/COX-2 expression, and blunting crystal-induced membrane lipid peroxidation; (3) European Phytotherapy: Standardized European phytotherapeutic agents—including *Herniaria glabra* (Rupturewort), *Solidago virgaurea* (Goldenrod), and *Equisetum arvense* (Horsetail)—contain specialized flavonoid glycosides and caffeic acid derivatives that exert diuretic, spasmolytic, and crystal-repelling actions on urothelial surfaces; (4) African Ethnopharmacology: Tropical medicinal plants such as *Phyllanthus amarus*/*niruri* (Chanca piedra), *Hibiscus sabdariffa* (Karkadeh), and *Enhydra fluctuans* (Chattaraj et al., 2023) modulate urinary pH, increase endogenous urinary citrate excretion, and inhibit Randall’s plaque calcification.

#### 2.3.4 Herbal formulations: multitarget synergy, strengths, and limitations

Polyherbal formulations and botanical extracts possess unique advantages in nephrolithiasis management, functioning both as expulsive/dissolution therapies and as preventive agents blunting recurrence through multi-target polypharmacology (Bao et al., 2021; Jiang et al., 2024; Zhao et al., 2023). Chemical classification of active constituents across studied herbal formulations highlights total flavonoids (e.g., quercetin, kaempferol, naringenin, luteolin) as the predominant pharmacologically active class (Figure 2) (Li S. et al., 2022; Lo et al., 2025). Large-scale epidemiological data from NHANES reveal a non-linear, J-shaped association between dietary flavanone intake and reduced kidney stone risk, providing clinical corroboration for flavonoid-mediated anti-urolithic effects (Gong et al., 2026).

**FIGURE 2:**
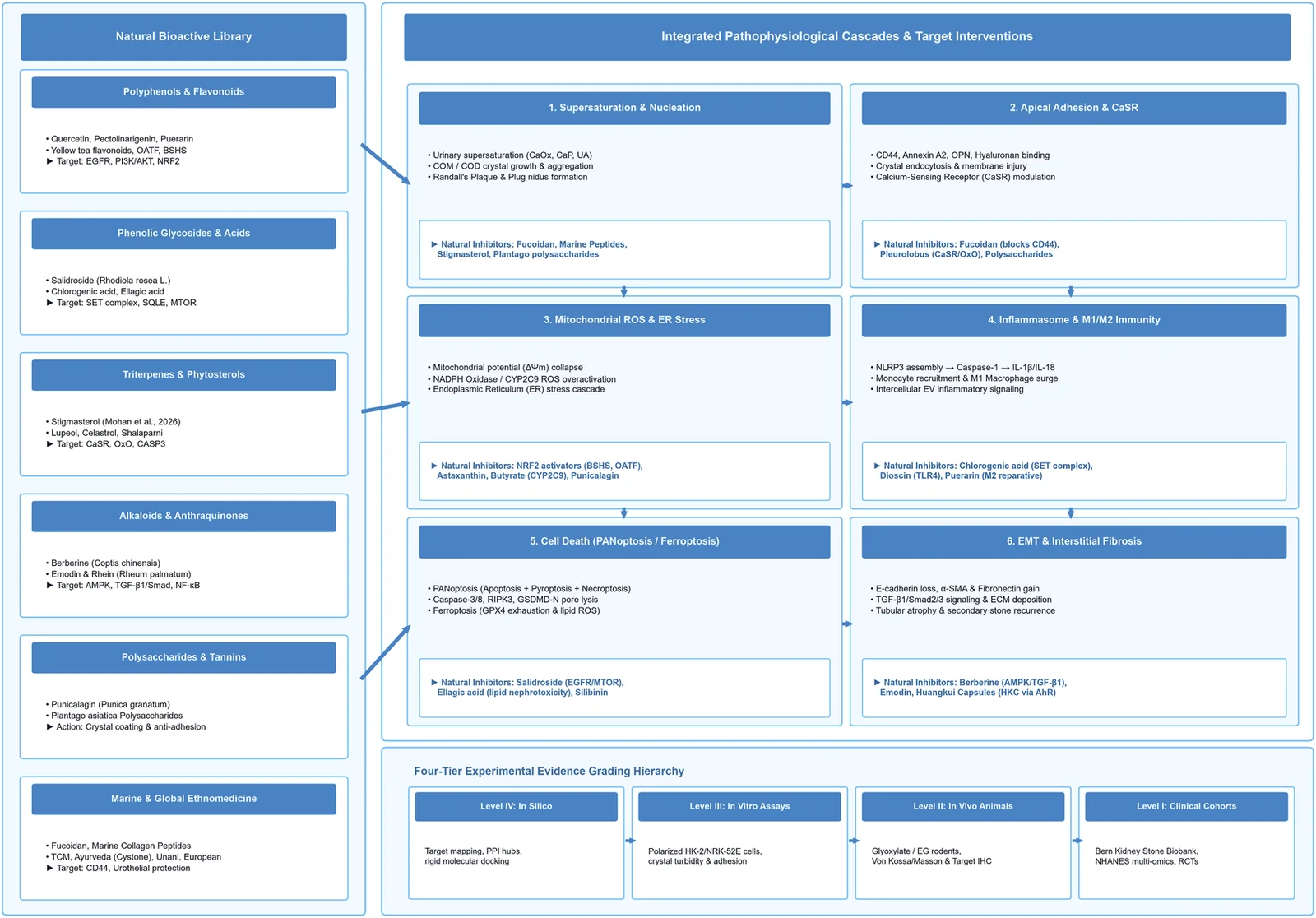
Comprehensive multi-level graphical summary synthesizing natural bioactive compounds, predicted molecular targets, signaling cascades, and biomineralization pathology in nephrolithiasis. Natural agents from global ethnomedicine (TCM, Ayurveda, Unani, European, African, and marine sources) modulate interconnected pathological axes: crystal nucleation and aggregation, apical CD44 receptor adhesion, mitochondrial oxidative burst, endoplasmic reticulum stress, NLRP3 inflammasome activation, macrophage M1/M2 polarization, PANoptosome assembly, and progressive tubulointerstitial fibrosis.

Mechanistically, herbal flavonoids exert synergistic protective actions across three interconnected pathological axes: (1) Antioxidant defense reinforcement: Excess CaOx crystallization stimulates ROS generation via NADPH oxidase and mitochondrial dysfunction (Khan, 2014; Ying et al., 2025). Total flavonoids from Orthosiphon aristatus (OATF) (Dai et al., 2025) act via the EGFR/PI3K/AKT axis to suppress ROS and malondialdehyde (MDA) while boosting superoxide dismutase (SOD) and glutathione peroxidase (GSH-PX) activities. Total flavonoids from Lysimachia christinae (Wang J. et al., 2021) and the multi-herb Bushen Huashi Decoction (BSHS) (Liu et al., 2023) activate the NRF2/ARE antioxidant response pathway, upregulating HO-1 and NQO-1 transcription and preserving tubular mitochondrial integrity; (2) Inflammatory cascade blunting: Crystals trigger NLRP3 inflammasome activation and pro-inflammatory cytokine release (IL-1β, IL-6, TNF-α) (Capolongo et al., 2023; Bai et al., 2025). Rhizoma Polygonati (Xu et al., 2024b) and Choerospondias axillaris (Qiu et al., 2025) extracts suppress NLRP3 and IL-1β expression in renal tissues, while seed extracts of Ficus religiosa (Pinipay et al., 2025) demonstrate potent anti-inflammatory efficacy in suppressing membrane destabilization (Antonelli et al., 2014); Anti-fibrotic modulation: Persistent nephrolithiasis induces chronic tubulointerstitial fibrosis. Huangkui Capsules (HKC) (Lin et al., 2025), containing rich flavonoid fractions, specifically activate the aryl hydrocarbon receptor (AhR) to suppress calcium deposition, tubular atrophy, and fibrogenesis. Similarly, yellow tea flavonoids (Su et al., 2023) target PPARG to inhibit epithelial-mesenchymal transition (EMT), curbing long-term fibrotic remodeling.

Critical Strengths and Methodological Limitations: The major strength of multi-target network pharmacology across diverse natural products lies in deciphering their holistic synergy—demonstrating how complex chemical mixtures simultaneously modulate crystal nucleation, interrupt the oxidative stress-inflammation-injury vicious cycle, and prevent downstream fibrotic remodeling. Nevertheless, substantial limitations persist across current studies: (1) *In vitro* model oversimplification: The vast majority of validations rely exclusively on 2D static monocultures of immortalized cell lines (HK-2 or NRK-52E) exposed to supra-physiological concentrations of oxalate/COM crystals, failing to simulate physiological urinary fluid shear stress, polarized epithelial transport, or dynamic immune-cell infiltration; (2) Pharmacokinetic (PK) and bioavailability disconnect: Most computational target predictions disregard whether oral administration achieves adequate free-drug concentrations in renal tissue or urine, creating a translational gap between micromolar *in silico* binding affinities and sub-nanomolar *in vivo* bioavailability; (3) Selective validation bias: Many studies computationally predict dozens of putative targets but validate only 1–2 arbitrarily selected proteins (e.g., AKT1 or TNF-α), leaving the broader network hypothesis unverified; (4) Quality standardization and batch variation in herbal extracts: Botanical extracts exhibit chemical heterogeneity depending on geographical origin and processing, requiring rigorous UHPLC-Q/TOF-MS chromatographic fingerprinting (Ding et al., 2026; Chen et al., 2021) to ensure reproducibility.

## 3 Critical appraisal of network pharmacology limitations and methodological quality

### 3.1 Core methodological bottlenecks in current network pharmacology

Despite its widespread adoption, network pharmacology in nephrolithiasis faces fundamental methodological bottlenecks that must be critically appraised to avoid misinterpreting *in silico* artifacts as true biological mechanisms (Nogales et al., 2022):

1. Target Prediction Bias and Database Discrepancies: Widely used target prediction engines (SwissTargetPrediction, PharmMapper, SEA) rely on distinct 2D/3D chemical similarity thresholds and training sets. Consequently, the same natural compound often yields highly discordant predicted target lists across different databases. Furthermore, databases exhibit significant latency in updating experimental pharmacology literature.
2. Publication Bias and Over-Representation of Ubiquitous “Hub” Genes: Disease-associated gene repositories (GeneCards, DisGeNET) are heavily skewed toward universally studied cancer and inflammation genes (e.g., TP53, VEGFA, AKT1, TNF, IL6, MAPK1). Topological degree centrality metrics invariably elevate these ubiquitous proteins to “core hub” status, creating a misleading appearance of disease-specific targeting in nephrolithiasis.
3. Arbitrary Pharmacokinetic Cutoffs (OB and DL): The standard criteria (oral bioavailability OB ≥ 30%, drug-likeness DL ≥ 0.18) inherited from Lipinski’s Rule of 5 are poorly suited for natural products. Many clinically potent anti-urolithic agents (e.g., berberine with OB < 5%, macromolecular polysaccharides, and ellagitannins) are routinely discarded by naive filtering, despite exerting profound actions *via* active gut microbial metabolites or local intestinal signaling.
4. Circular Virtual Validation and Docking Confirmation Bias: A prevalent circular reasoning flaw occurs when virtual molecular docking is performed against the very same target proteins that were initially predicted based on structural similarity to known ligands. Furthermore, standard rigid-body docking neglects receptor conformational dynamics and water-mediated hydrogen bonding networks, generating false-positive binding energies.
5. Disregard for Pharmacokinetics, Biotransformation, and Renal Bioavailability: *In silico* network models routinely assume that parent phytochemicals reach intrarenal target receptors intact at micromolar concentrations. In reality, most polyphenols and flavonoids undergo extensive Phase II glucuronidation, sulfation, and methylation in the liver and gut. Without measuring actual free-drug concentrations (fu) and active metabolite profiles in renal tissue or urine, predicted network mechanisms remain hypothetical.
6. Confirmation Bias, Selective Reporting, and Handling of Discordant Negative Data: Another critical methodological vulnerability in contemporary network pharmacology is the pervasive influence of confirmation bias and selective reporting. When experimental validations, pathway enrichment analyses (e.g., GSEA or KEGG), or multi-omics profiles yield negative, inconsistent, or non-statistically significant results relative to initial computational predictions, researchers frequently engage in *post hoc* selective reporting or force-fit interpretations to reconcile the discrepancies. To establish network pharmacology as a rigorous empirical science, future studies must adhere to transparent reporting standards (such as the CONSERVE and FAIR guidelines for systems biology) (Shin et al., 2023). Specifically: (1) unvalidated candidate targets and non-significant pathways should be fully disclosed rather than omitted; and (2) incongruent gene expression directions between computational predictions and empirical transcriptomic/proteomic datasets should be treated as informative biological insights—such as compensatory feedback loops, post-translational modifications, or cell-specific microenvironment effects—rather than forced into pre-existing paradigms. Embracing negative data and reporting discordant findings will eliminate false-positive translational targets and foster realistic clinical drug discovery.

### 3.2 Experimental evidence grading and validation rigor

To overcome descriptive reporting, future network pharmacology studies must adhere to a structured four-tier evidence-grading hierarchy: (1) Level I (Clinical Evidence): Direct verification of target expression, biomarker alteration, or stone recurrence prevention in human randomized controlled trials (RCTs) or longitudinal prospective cohorts; (2) Level II (*In Vivo* Animal Evidence): Validation in established stone-forming rodent models (glyoxylate, ethylene glycol, or L-hydroxyproline administration) demonstrating histopathological attenuation of crystal deposition, preservation of renal function, and target protein modulation via western blot and immunohistochemistry; (3) Level III (*In Vitro* Cellular Evidence): Mechanistic assays in polarized renal tubular cells or co-culture systems evaluating crystal adhesion, ROS production, mitochondrial membrane potential, and target knockdown/overexpression; (4) Level IV (*In Silico* Computational Prediction): Pure bioinformatics target mapping and molecular docking without biological corroboration. As detailed in Table 2, studies combining Level II and Level III validation provide the minimal threshold for scientific credibility (incorporating established systems biology reporting standards) (Shin et al., 2023).

## 4 Clinical translation, regulatory challenges, and future directions

### 4.1 Clinical translation, standardization, and herb-drug interactions

Translating natural anti-urolithic candidates from computational networks into clinical practice requires overcoming significant pharmaceutical and regulatory hurdles: (1) Chemical Standardization and Quality Control: Multi-component botanical formulations require rigorous chromatographic fingerprinting (UHPLC-Q/TOF-MS) and quantitative multi-ingredient marker assays to ensure batch-to-batch consistency and regulatory compliance (e.g., US FDA botanical drug guidance, EMA herbal monographs); (2) Formulation Engineering and Renal-Targeted Delivery: Poor aqueous solubility and low renal bioavailability of hydrophobic phytochemicals (e.g., celastrol, silibinin) can be overcome through advanced drug delivery systems, including lipid nanoparticles, polymeric micelles, and renal tubule-targeted peptide conjugates; (3) Herb-Drug Interactions (HDIs) and Toxicology: Natural products frequently modulate cytochrome P450 enzymes (CYP3A4, CYP2C9) and drug transporters (P-glycoprotein), presenting risks of adverse interactions when co-administered with conventional cardiovascular, immunosuppressive, or urological drugs. Rigorous safety profiling and Phase I clinical trials are imperative.

### 4.2 Emerging translational paradigms and future roadmap

#### 4.2.1 Multi-omics and single-cell RNA sequencing integration

Integrating multi-omics data (GWAS genomics, transcriptomics, proteomics, metabolomics, gut microbiomics) provides high-confidence multilayered networks (Zhang et al., 2025; Xu et al., 2022). Single-cell RNA sequencing (scRNA-seq) resolves cell type-specific compound actions (Xu et al., 2025b)—clarifying whether natural agents directly protect proximal tubular epithelial subsets (mitigating oxidative stress, ferroptosis, or EMT) or reprogram intrarenal immune cell dynamics (suppressing M1 macrophage polarization and recruiting reparative M2 macrophages) (Jahangir et al., 2022).

#### 4.2.2 Humanized 3D organoids, spatial omics, and clinical biobank reverse validation

Patient-Derived Organoids (PDOs), particularly renal tubuloids co-cultured with autologous immune cells under dynamic fluid shear stress, provide physiologically relevant microenvironments to evaluate crystal endocytosis and barrier integrity in real time (Lin et al., 2024). Furthermore, Spatial Transcriptomics (ST) coupled with multiplex Immunohistochemistry (mIHC) maps predicted targets directly within crystal injury niches (Wang et al., 2023; Sun et al., 2026; Ng-Blichfeldt et al., 2024). Finally, reverse validation against longitudinal clinical registries (Bern Kidney Stone Registry, NHANES) and stone specimen matrix proteomes firmly anchors computational predictions in human clinical reality (Zhou et al., 2025; Anderegg et al., 2024; Jia et al., 2025).

#### 4.2.3 Future outlook: machine learning and AI-assisted target discovery

As summarized in Table 3, machine learning algorithms upgrade network pharmacology from heuristic models into quantitative predictive frameworks (Cui et al., 2025; Liu et al., 2026; Huo et al., 2022): (1) L1-regularized LASSO regression eliminates false-positive candidate genes via sparse feature selection Peng et al. (2023); (2) Random Forest and Boruta capture non-linear synergistic multi-component interactions (Liu et al., 2026; Huang et al., 2025); (3) SVM-RFE extracts minimal optimal core target panels, minimizing unnecessary experimental validation costs (Peng et al. 2024); (4) Graph Neural Networks (GCN/GAT) model dynamic heterogeneous “Drug-Target-Pathway” graphs to predict unknown interactions and herb compatibility (Guo et al., 2024; Gu et al., 2025; Gilligan et al., 2023; Kesimoglu and Bozdag, 2024); and (5) AlphaFold and deep docking enable structural prediction and high-throughput virtual screening for previously uncharacterized crystal-binding targets (Jumper et al., 2021).

**TABLE 3:** Potential application scenarios, computational principles, and methodological advantages of advanced machine learning algorithms in target screening for kidney stone research.

ML algorithm	Core computational principles	Application scenarios in nephrolithiasis target screening	Methodological advantages over static networks	Key references
LASSO Regression	Applies L1 regularization penalty to shrink non-essential target coefficients to zero, achieving sparse feature selection	Filters core target genes from initial compound-disease candidate intersections, eliminating redundant background nodes	Overcomes high false-positive rates and non-specificity inherent in traditional degree/betweenness centrality metrics	Wang et al. (2023); Peng et al. (2023)
Random Forest and Boruta	Evaluates mean decrease in Gini impurity across ensemble decision trees to rank target feature importance	Ranks functional importance of targets modulating crystal nucleation, oxidative stress, and inflammatory cascades	Captures complex non-linear synergistic interactions between multi-component phytomedicines and targets without single-model bias	Huang et al. (2025); Liu et al. (2026)
SVM-RFE	Combines maximal-margin hyperplanes with backward feature elimination to identify minimal optimal feature subsets	Extracts the minimal core target panel discriminating stone formation or treatment response in damaged renal epithelial cells	Minimizes necessary experimental validation targets while preserving high predictive classification accuracy	Peng et al. (2024)
XGBoost and LightGBM	Implements gradient boosted decision trees with regularization to prevent overfitting and capture non-linear patterns	Predicts active compound renal targeting, oral bioavailability, and compound-target binding probability	Replaces arbitrary, static OB/DL thresholds with quantitative confidence scoring for compound screening	Huo et al. (2022); Liu et al. (2026)
Graph Neural Networks (GCN/GAT)	Transmits node representations across heterogeneous graph topologies using graph attention and message-passing mechanisms	Models dynamic multi-layer “drug-target-pathway” networks, predicts unknown drug-target interactions (DTIs), and evaluates herb compatibility	Overcomes static undirected network limits by incorporating dynamic directional topology and edge weight heterogeneity	Guo et al. (2024); Gu et al. (2025); Kesimoglu and Bozdag (2024)
Deep Docking and AlphaFold	Integrates neural network-predicted 3D target structures with deep learning binding affinity scoring	Enables 3D structure prediction and high-throughput virtual screening for uncharacterized kidney stone target proteins	Bypasses rigid receptor constraints and high computational costs of flexible docking, accelerating natural compound screening	Jumper et al. (2021)

## 5 Conclusion

Nephrolithiasis remains a major global health challenge driven by complex biomineralization and cell-injury cascades with high recurrence rates. Network pharmacology provides a powerful systems biology methodology to decode the multi-target, multi-pathway polypharmacology of natural bioactive products from diverse global ethnomedicinal traditions—including Traditional Chinese Medicine, Indian Ayurveda, Persian, European, African, and marine resources. While recent studies (2022–2026) have uncovered significant mechanisms across classical antioxidant/anti-inflammatory pathways and newly identified axes (PANoptosis, lipid nephrotoxicity, ferroptosis, CYP2C9 modulation), future progress demands critical vigilance against target prediction biases, unverified pharmacokinetic assumptions, and circular validation flaws. By establishing standardized evidence-grading frameworks and integrating single-cell transcriptomics, humanized 3D organoid co-cultures, spatial multi-omics, clinical biobank reverse validation, and machine learning feature selection, natural product research will successfully transition from descriptive network mapping toward precision translational pharmacology for kidney stone prevention.

## References

[B1] Ahmed S. Hasan M. M. U. Khan H. Mahmood Z. A. Patel S. (2018). The mechanistic insight of polyphenols in calcium oxalate urolithiasis mitigation. Biomed. Pharmacother. 106, 1292–1299. 10.1016/j.biopha.2018.07.080 30119199

[B2] Anderegg M. A. Olinger E. G. Bargagli M. Geraghty R. Taylor L. Nater A. (2024). Prevalence and characteristics of genetic disease in adult kidney stone formers. Nephrol. Dial. Transpl. 39, 1426–1441. 10.1093/ndt/gfae074 PMC11483609 38544324

[B3] Antonelli J. A. Maalouf N. M. Pearle M. S. Lotan Y. (2014). Use of the National Health and Nutrition Examination Survey to calculate the impact of obesity and diabetes on cost and prevalence of urolithiasis in 2030. Eur. Urol. 66, 724–729. 10.1016/j.eururo.2014.06.036 25015037 PMC4227394

[B4] Bai J. Lu T. Zhao Z. Wei F. He K. He X. (2025). The role of inflammation and ROS in CaOx kidney stones. Eur. J. Med. Res. 30, 1224. 10.1186/s40001-025-03469-4 41372793 PMC12690943

[B5] Bao C. Ni T. Shao S. Zhuang X. Zhuo X. Wan X. (2021). Complex renal calculi treated with traditional Chinese medicine Paishi decoction combined with laparoscopic ureterectomy. Pak J. Pharm. Sci. 34, 2423–2427. 10.36721/PJPS.2021.34.6.SP.2423-2427.1 35039254

[B6] Bhura N. Gupta J. (2025). Exploring phytochemical candidates against nephrolithiasis via similarity searching, network pharmacology, and docking studies. Biochem. Biophys. Res. Commun. 771, 151975. 10.1016/j.bbrc.2025.151975 40393161

[B7] Capolongo G. Ferraro P. M. Unwin R. (2023). Inflammation and kidney stones: cause and effect? Curr. Opin. Urol. 33, 129–135. 10.1097/mou.0000000000001066 36562282

[B8] Chaiyarit S. Thongboonkerd V. (2020). Mitochondrial dysfunction and kidney stone disease. Front. Physiol. 11, 566506. 10.3389/fphys.2020.566506 33192563 PMC7606861

[B9] Chattaraj B. Khanal P. Nandi A. Das A. Sharma A. Mitra S. (2023). Network pharmacology and molecular modelling study of enhydra fluctuans for the prediction of the molecular mechanisms involved in the amelioration of nephrolithiasis. J. Biomol. Struct. Dyn. 41, 15400–15410. 10.1080/07391102.2023.2189476 36914227

[B10] Chen W. Si Y. Cheng J. Ding J. Zhao H. Liu W. (2021). Metabolic and network pharmacological analyses of the therapeutic effect of Grona styracifolia on calcium oxalate-induced renal injury. Front. Pharmacol. 12, 652989. 10.3389/fphar.2021.652989 34248618 PMC8267468

[B11] Cui G. Li M. Guo W. Gao M. Zhu Q. Liao J. (2025). AI driven network pharmacology: multi-scale mechanisms of traditional Chinese medicine from molecular to patient analysis. Comput. Struct. Biotechnol. J. 27, 5087–5104. 10.1016/j.csbj.2025.11.016 PMC12663848 41322006

[B12] Dai S. Fang Q. Li H. Y. Sun R. Zhang H. Y. Wu W. (2025). Network pharmacology and experimental validation to elucidate the pharmacological mechanisms of OATF against kidney stones. Front. Pharmacol. 16, 1575270. 10.3389/fphar.2025.1575270 40432899 PMC12106310

[B13] Ding J. Xu F. Chen D. Xi J. Gao F. Chen L. (2026). Qing Hua Yu Du formula ameliorates alcoholic hepatic fibrosis by regulating MAPK/TLR4-MyD88 inflammatory pathways, restoring hepatic metabolism and modulating gut microbiota. Phytomedicine 152, 157830. 10.1016/j.phymed.2026.157830 41570783

[B14] Evan A. P. Lingeman J. E. Coe F. L. Parks J. H. Bledsoe S. B. Shao Y. (2003). Randall's plaque of patients with nephrolithiasis begins in basement membranes of thin loops of Henle. J. Clin. Invest 111, 607–616. 10.1172/JCI17038 12618515 PMC151900

[B15] Gilligan L. P. J. Cobelli M. Taufour V. Sanvito S. (2023). A rule-free workflow for the automated generation of databases from scientific literature. NPJ Comput. Mater 9, 222. 10.1038/s41524-023-01171-9 38666056 PMC11041762

[B16] Gong Y. Yang Y. Miao L. Zhong M. Xia Q. Chang J. (2026). Flavonoid intake and kidney stones: an exposome-based cross-sectional study. J. Nutr. Biochem. 147, 110117. 10.1016/j.jnutbio.2025.110117 40967377

[B17] Gu L. Ma Y. Liu S. Zhang Q. Zhang Q. Ma P. (2025). Prediction of herbal compatibility for colorectal adenoma treatment based on graph neural networks. Chin. Med. 20, 31. 10.1186/s13020-025-01082-5 40045358 PMC11881240

[B18] Guo X. Zhao X. Lu X. Zhao L. Zeng Q. Chen F. (2024). A deep learning-driven discovery of berberine derivatives as novel antibacterial against multidrug-resistant Helicobacter pylori. Signal Transduct. Target Ther. 9, 183. 10.1038/s41392-024-01895-0 PMC11228022 38972904

[B19] Hou X. Meng H. Ying D. Tian R. Guo X. Li B. (2025). Decoding coffee's chlorogenic acid: genetic-network pharmacology insights on kidney stones. Urolithiasis 53, 124. 10.1007/s00240-025-01776-3 40542847

[B20] Huang S. Zhang L. Liu X. (2025). Bioinformatics approach to identifying molecular targets of isoliquiritigenin affecting chronic obstructive pulmonary disease: a machine learning pharmacology study. Int. J. Mol. Sci. 26, 3907. 10.3390/ijms26083907 40332792 PMC12027559

[B21] Huo M. Peng S. Li J. Zhang Y. Qiao Y. (2022). A discovery strategy for active compounds of Chinese medicine based on the prediction model of compound-disease relationship. J. Oncol. 2022, 8704784. 10.1155/2022/8704784 35847368 PMC9286898

[B22] Jahangir M. Yazdani O. Kahrizi M. S. Soltanzadeh S. Javididashtbayaz H. Mivefroshan A. (2022). Clinical potential of PD-1/PD-L1 blockade therapy for renal cell carcinoma (RCC): a rapidly evolving strategy. Cancer Cell Int. 22, 401. 10.1186/s12935-022-02816-3 36510217 PMC9743549

[B23] Jia Y. Han M. Ge H. Qiao J. Chen R. Li C. (2025). Dysbiosis of gut and urinary microbiota in urolithiasis patients and post-surgical cases. Front. Cell Infect. Microbiol. 15, 1633783. 10.3389/fcimb.2025.1633783 40880629 PMC12380916

[B24] Jiang Q. Dong C. He Z. Wang Y. Jiang R. Liao W. (2024). Research landscape and pharmacological mechanisms of traditional Chinese medicines in treating and preventing urolithiasis: unearthing an anti-urolithic treasure trove. J. Ethnopharmacol. 334, 118502. 10.1016/j.jep.2024.118502 38950794

[B25] Jong B. E. Wu H. C. Chen W. C. Wu W. J. Li C. C. Lu Y. H. (2025). Kidney stone history and survival outcomes in upper tract urothelial carcinoma. JAMA Netw. Open 8, e2541054. 10.1001/jamanetworkopen.2025.41054 PMC12584037 41182764

[B26] Jumper J. Evans R. Pritzel A. Green T. Figurnov M. Ronneberger O. (2021). Highly accurate protein structure prediction with AlphaFold. Nature 596, 583–589. 10.1038/s41586-021-03819-2 34265844 PMC8371605

[B27] Kesimoglu Z. N. Bozdag S. (2024). Fusing multiplex heterogeneous networks using graph attention-aware fusion networks. Sci. Rep. 14, 29119. 10.1038/s41598-024-78555-4 39582056 PMC11586420

[B28] Khan S. R. (2014). Reactive oxygen species, inflammation and calcium oxalate nephrolithiasis. Transl. Androl. Urol. 3, 256–276. 10.3978/j.issn.2223-4683.2014.06.04 25383321 PMC4220551

[B29] Khan S. R. Pearle M. S. Robertson W. G. Gambaro G. Canales B. K. Doizi S. (2016). Kidney stones. Nat. Rev. Dis. Prim. 2, 16008. 10.1038/nrdp.2016.8 27188687 PMC5685519

[B30] Khan S. R. Canales B. K. Morrow P. R. (2021). Randall's plaque and calcium oxalate nephrolithiasis: a new look at an old hypothesis. Clin. Kidney J. 14, 1751–1758. 10.1093/ckj/sfab021

[B31] Lai Y. Zheng H. Sun X. Lin J. Li Q. Huang H. (2022). The advances of calcium oxalate calculi associated drugs and targets. Eur. J. Pharmacol. 935, 175324. 10.1016/j.ejphar.2022.175324 36257382

[B32] Li M. T. Liu L.-L. Zhou Q. Huang L.-X. Shi Y.-X. Hou J.-B. (2022a). Exerts protective effects against the calcium oxalate-induced renal injury via ellagic acid. Front. Pharmacol. 13, 891788. 10.3389/fphar.2022.891788 PMC9400657 36034880

[B33] Li S. Macaringue E. G. Zhou D. Shi P. Tang W. Gong J. (2022b). Discovering inhibitor molecules for pathological crystallization of CaOx kidney stones from natural extracts of medical herbs. J. Ethnopharmacol. 284, 114733. 10.1016/j.jep.2021.114733 34644589

[B34] Li G. Song Z. Yao J. Chen Y. Xu Y. Hao Z. (2026). Salidroside attenuates calcium oxalate-induced renal injury via suppression of PANoptosis. Urolithiasis 54, 25. 10.1007/s00240-025-01927-6 41483174

[B35] Liang H. Xu Y. Sun S. Chen Y. Wang W. Hao Z. (2024). Based on network pharmacology, the mechanism of dioscin in alleviating renal tubular epithelial cell injury induced by calcium oxalate crystals was explored. Urolithiasis 53, 3. 10.1007/s00240-024-01673-1 39666011

[B36] Lin S. Ren Z. Li L. Xia S. Yang R. Ye H. (2024). LncRNA AP001007 protects human renal tubular epithelial HK-2 cells and kidney organoids from LPS-induced injury. Sci. Rep. 14, 28578. 10.1038/s41598-024-79367-2 39562779 PMC11577071

[B37] Lin H. Li X. Shen A. Yin W. (2025). Pivotal role of AhR protein in the therapeutic efficacy of HuangKui capsule against calcium oxalate-induced kidney damage: an integrated study of network pharmacology and experimental verification. Int. J. Biol. Macromol. 319, 145288. 10.1016/j.ijbiomac.2025.145288 40550434

[B38] Liu H. Cao M. Jin Y. Jia B. Wang L. Dong M. (2023). Network pharmacology and experimental validation to elucidate the pharmacological mechanisms of Bushen Huashi decoction against kidney stones. Front. Endocrinol. (Lausanne) 14, 1031895. 10.3389/fendo.2023.1031895 36864834 PMC9971497

[B39] Liu T. Lyu C. Wang X. (2026). Resveratrol ameliorates sepsis-induced immune dysfunction by targeting GADD45A to maintain hematopoietic stem cell homeostasis: a predictive study integrating ensemble machine learning, single-cell transcriptomics, and molecular dynamics simulation. Int. Immunopharmacol. 176, 116470. 10.1016/j.intimp.2026.116470 41806693

[B40] Lo C. Y. Z. Khor Q. H. Abdullatif V. A. Delgado C. Lu Y. Katz J. (2025). Systematic review of pharmacological, complementary, and alternative therapies for the prevention of calcium oxalate stones. Asian J. Urol. 12, 169–188. 10.1016/j.ajur.2024.04.006 40458577 PMC12126926

[B41] Lobine D. Ahmed S. Aschner M. Khan H. Mirzaei H. Mahomoodally M. F. (2020). Antiurolithiatic effects of pentacyclic triterpenes: the distance traveled from therapeutic aspects. Drug Dev. Res. 81, 671–684. 10.1002/ddr.21670 32314397

[B42] Maciejewska-Turska M. Georgiev M. I. Kai G. Sieniawska E. (2025). Advances in bioinformatic methods for the acceleration of the drug discovery from nature. Phytomedicine 139, 156518. 10.1016/j.phymed.2025.156518 40010031

[B43] Mohan P. K. Ajeesh Krishna T. Stephy P. Antony Ceasar S. Adarsh Krishna T. Annamalai A. (2025). Anti-urolithiatic potential of *Pleurolobus gangeticus* against calcium-sensing receptor and oxalate oxidase targets. Results Chem. 18, 102906. 10.1016/j.rechem.2025.102906

[B44] Mohan P. K. Adarsh Krishna T. P. Stephy P. P. Ajeesh Krishna T. P. Thirumurugan A. Annamalai A. (2026). Stigmasterol as a potential anti-urolithiatic agent: *in vitro* evaluation and computational insights into renal targets. Syst. Ethnopharmacol. Sustain Bioresour. 1, 100002. 10.1016/j.sesb.2026.100002

[B45] Ng-Blichfeldt J. P. Stewart B. J. Clatworthy M. R. Williams J. M. Röper K. (2024). Identification of a core transcriptional program driving the human renal mesenchymal-to-epithelial transition. Dev. Cell 59, 595–612.e598. 10.1016/j.devcel.2024.01.011 38340720 PMC7616043

[B46] Nogales C. Mamdouh Z. M. List M. Kiel C. Casas A. I. Schmidt H. H. (2022). Network pharmacology: curing causal mechanisms instead of treating symptoms. Trends Pharmacol. Sci. 43, 136–150. 10.1016/j.tips.2021.11.004 34895945

[B47] Noor F. Tahir ul Qamar M. Ashfaq U. A. Albutti A. Alwashmi A. S. S. Aljasir M. A. (2022). Network pharmacology approach for medicinal plants: review and assessment. Pharm. (Basel) 15, 572. 10.3390/ph15050572 PMC9143318 35631398

[B48] Peng W. Yang Y. Lu H. Shi H. Jiang L. Liao X. (2024). Network pharmacology combines machine learning, molecular simulation dynamics and experimental validation to explore the mechanism of acetylbinankadsurin A in the treatment of liver fibrosis. J. Ethnopharmacol. 323, 117682. 10.1016/j.jep.2023.117682 38169205

[B49] Peng J. Yang K. Tian H. Lin Y. Hou M. Gao Y. (2023). The mechanisms of Qizhu Tangshen formula in the treatment of diabetic kidney disease: network pharmacology, machine learning, molecular docking and experimental assessment. Phytomedicine 108, 154525. 10.1016/j.phymed.2022.154525 36413925

[B50] Pinipay F. Rokkam R. Botcha S. Tamanam R. R. (2025). Metabolomic and network pharmacology insights into the anti-inflammatory and anti-urolithiatic potential of Ficus religiosa seeds. Sci. Rep. 15, 39098. 10.1038/s41598-025-26906-0 41203807 PMC12594937

[B51] Qiu M. Li S. Zhang Y. Yan J. Qin S. Yin X. (2025). Multi—dimensional mechanism analysis of Choerospondias axillaris (Roxb.) Burtt et Hill in treating kidney stones: network pharmacology, molecular docking and *in vitro* experimental verification. Front. Pharmacol. 16, 1501386. 10.3389/fphar.2025.1501386 40331191 PMC12053011

[B52] Shin J. Porubsky V. Carothers J. Sauro H. M. (2023). Standards, dissemination, and best practices in systems biology. Curr. Opin. Biotechnol. 81, 102922. 10.1016/j.copbio.2023.102922 37004298 PMC10435326

[B53] Sorokin I. Mamoulakis C. Miyazawa K. Rodgers A. Talati J. Lotan Y. (2017). Epidemiology of stone disease across the globe. World J. Urol. 35, 1301–1320. 10.1007/s00345-017-2008-6 28213860

[B54] Srivastava N. K. Panwar V. K. Mittal A. Mandal A. K. Agarwal H. (2025). Long-term outcomes of percutaneous nephrolithotomy in 502 chronic kidney disease patients: insights from a tertiary center. World J. Urol. 43, 422. 10.1007/s00345-025-05804-0 40627037

[B55] Su M. Sang S. Liang T. Li H. (2023). PPARG: a novel target for yellow tea in kidney stone prevention. Int. J. Mol. Sci. 24, 11955. 10.3390/ijms241511955 37569334 PMC10418378

[B56] Sun L. Li R. Zhang K. Gao W. (2026). Mechanistic study on comorbidities of kidney stones and sepsis using transcriptome sequencing combined with single-cell spatial transcriptome sequencing. Med. Baltim. 105, e49779. 10.1097/md.0000000000049779 PMC13384656 42469985

[B57] Wang Z. Zhang Y. Zhang J. Deng Q. Liang H. (2021a). Recent advances on the mechanisms of kidney stone formation (review). Int. J. Mol. Med. 48, 149. 10.3892/ijmm.2021.4982 PMC8208620 34132361

[B58] Wang J. Chen J. J. Huang J. H. Lv B. D. Huang X. J. Hu Q. (2021b). Protective effects of total flavonoids from Lysimachia christinae on calcium oxalate-induced oxidative stress in a renal cell line and renal tissue. Evid. Based Complement. Altern. Med. 2021, 6667902–6667911. 10.1155/2021/6667902 PMC8481038 34603474

[B59] Wang Z. Deng Q. Gu Y. Li M. Chen Y. Wang J. (2023). Integrated single-nucleus sequencing and spatial architecture analysis identified distinct injured-proximal tubular types in calculi rats. Cell Biosci. 13, 92. 10.1186/s13578-023-01041-3 37208718 PMC10197242

[B60] Xu X. Chen J. Lv H. Xi Y. Ying A. Hu X. (2022). Molecular mechanism of Pyrrosia lingua in the treatment of nephrolithiasis: network pharmacology analysis and *in vivo* experimental verification. Phytomedicine 98, 153929. 10.1016/j.phymed.2022.153929 35104754

[B61] Xu Z. Yao X. Duan C. Liu H. Xu H. (2023). Metabolic changes in kidney stone disease. Front. Immunol. 14, 1142207. 10.3389/fimmu.2023.1142207 37228601 PMC10203412

[B62] Xu Y. Liang H. Mao X. Song Z. Shen X. Ge D. (2024a). Puerarin alleviates apoptosis and inflammation in kidney stone cells via the PI3K/AKT pathway: network pharmacology and experimental verification. J. Cell Mol. Med. 28, e70180. 10.1111/jcmm.70180 PMC11512754 39462270

[B63] Xu Y. Liang H. Mao X. Chen Y. Hou B. Hao Z. (2024b). Molecular mechanism of rhizoma polygonati in the treatment of nephrolithiasis: network pharmacology analysis and *in vivo* experimental verification. Urolithiasis 52, 35. 10.1007/s00240-024-01533-y 38376588

[B64] Xu Y. Liang H. Xia K. Yao J. Chen Y. Hou B. (2025a). Umbelliferone attenuates calcium oxalate crystal-induced renal injury and inflammation by attenuating autophagy through the PI3K/AKT pathway. Int. Immunopharmacol. 150, 114250. 10.1016/j.intimp.2025.114250 39952007

[B65] Xu Y. Zheng Z. Oswald M. S. Cheng G. Liu J. Zhai Q. (2025b). Single-cell RNA sequencing delineates renal anti-fibrotic mechanisms mediated by TRPC6 inhibition. Adv. Sci. (Weinh) 12, e01175. 10.1002/advs.202501175 PMC12412467 40525246

[B66] Yao R. Pan J. S. He R. B. Hou B. B. Suo X. G. Li G. X. (2024). Pectolinarigenin alleviates calcium oxalate-induced renal inflammation and oxidative stress by binding to HIF-1α. Int. Immunopharmacol. 143, 113284. 10.1016/j.intimp.2024.113284 39378657

[B67] Ying X. Chen Y. Hao Z. Liu H. (2025). The significance of reactive oxygen species in the formation of calcium oxalate stones and the protective effects of antioxidants on the kidneys. Front. Immunol. 16, 1540075. 10.3389/fimmu.2025.1540075 40469275 PMC12133935

[B68] Zhang X. Jing Q. Yao W. Zhang B. Yuan X. (2025). Unraveling the role of gut microbiota on the formation of nephrolithiasis: insights from integrated analysis of GWAS, single-cell transcriptomics, bulk RNA sequencing and network pharmacology. Urolithiasis 53, 138. 10.1007/s00240-025-01809-x 40664942 PMC12263484

[B69] Zhao L. Zhang H. Li N. Chen J. Xu H. Wang Y. (2023). Network pharmacology, a promising approach to reveal the pharmacology mechanism of Chinese medicine formula. J. Ethnopharmacol. 309, 116306. 10.1016/j.jep.2023.116306 36858276

[B70] Zhou Z. Zhou X. Zhang Y. Yang Y. Wang L. Wu Z. (2023). Butyric acid inhibits oxidative stress and inflammation injury in calcium oxalate nephrolithiasis by targeting CYP2C9. Food Chem. Toxicol. 178, 113925. 10.1016/j.fct.2023.113925 37414240

[B71] Zhou L. T. Stark I. W. Jayachandran M. Harris P. C. Rule A. D. Koo K. (2025). A multiomics study of circulating proteins and kidney stone risk. J. Am. Soc. Nephrol. 36, 2456–2468. 10.1681/asn.0000000768 40577066 PMC12677822

